# Is It Really a Matter of Simple Dualism? Corticotropin-Releasing Factor Receptors in Body and Mental Health

**DOI:** 10.3389/fendo.2013.00028

**Published:** 2013-03-12

**Authors:** Donny Janssen, Tamás Kozicz

**Affiliations:** ^1^Department of Cellular Animal Physiology, Donders Institute for Brain, Cognition and BehaviorNijmegen, Netherlands; ^2^Department of Anatomy, Donders Institute for Brain, Cognition and BehaviorNijmegen, Netherlands; ^3^Human Genetics Center, Tulane UniversityNew Orleans, LA, USA

**Keywords:** CRFR1, CRFR2, CRF, Urocortins, stress, anxiety, depression, HPA-axis

## Abstract

Physiological responses to stress coordinated by the hypothalamo-pituitary-adrenal axis are concerned with maintaining homeostasis in the presence of real or perceived challenges. Regulators of this axis are corticotrophin releasing factor (CRF) and CRF related neuropeptides, including urocortins 1, 2, and 3. They mediate their actions by binding to CRF receptors (CRFR) 1 and 2, which are located in several stress-related brain regions. The prevailing theory has been that the initiation of and the recovery from an elicited stress response is coordinated by two elements, viz. the (mainly) opposing, but well balanced actions of CRFR1 and CRFR2. Such a dualistic view suggests that CRF/CRFR1 controls the initiation of, and urocortins/CRFR2 mediate the recovery from stress to maintain body and mental health. Consequently, failed adaptation to stress can lead to neuropathology, including anxiety and depression. Recent literature, however, challenges such dualistic and complementary actions of CRFR1 and CRFR2, and suggests that stress recruits CRF system components in a brain area and neuron specific manner to promote adaptation as conditions dictate.

“The idea would be not to abolish the CRFR1 receptor’s response to the brain’s stress hormone but to bring it into the normal range so that it would have appropriate levels of anxiety and stress as conditions dictate.”

*Wylie W. Vale (1941–2012)*

## Introduction

The concept of stress and adaptation was first observed in 1936 and further defined in 1951 (Selye, 1936, 1951). After exposure to a stressor, corticotrophin releasing factor (CRF) is released by endocrine cells of the paraventricular nucleus (PVN) of the hypothalamus into the portal vessels thereby stimulating the release of adrenocorticotropic hormone (ACTH) by the anterior pituitary. Subsequently, circulating ACTH stimulates secretion of glucocorticoids from the adrenal gland (Vale et al., 1981). In the 1980s, the group of Vale isolated the 41-residue CRF peptide from the ovine hypothalamus (Vale et al., 1981) and throughout the decades, many members of the CRF family have been identified, including urocortin 1 (Ucn1), urocortin 2 (Ucn2), and urocortin 3 (Ucn3). The urocortins differ in their tissue distribution and receptor pharmacology (Vaughan et al., 1995; Hsu and Hsueh, 2001; Lewis et al., 2001; Reyes et al., 2001). First shown to be important regulators of the endocrine stress response, the CRF family of neuropeptides is now known to play a role in diverse roles of homeostatic balance, important in mobilization of resources and behaviors during stress (Bale and Vale, 2004). In addition, members of the CRF peptide family play a role in regulation of food intake and satiety, as well as gastrointestinal tract motility, vascular tone, and development, and also acoustic and cardiac function (Heinrichs et al., 1992; Spina et al., 1996; Parkes et al., 1997; Okosi et al., 1998; Koob and Heinrichs, 1999; Maillot et al., 2000; Terui et al., 2001; Vetter et al., 2002; Inoue et al., 2003).

The actions of these peptides are initiated by binding and activating G-protein coupled receptors, CRFR1 and CRFR2, which display distinct affinity for members of the CRF peptide family, with CRF and Ucn1 binding to both receptors, while Ucn2 and 3 selectively bind to CRFR2 (Vaughan et al., 1995; Lewis et al., 2001). Besides CRF receptors (CRFRs) and their cognate ligands, another peptide that plays an important role in the neurobiology of the CRF family of neuropeptides is CRF binding protein (CRF-BP), a 37 kDa N-linked glycoprotein which binds both CRF and Ucn1 with high affinity (Orth and Mount, 1987; Boorse et al., 2005).

In this review, we highlight evidence supporting the notion of a dualistic action of CRFR1 and CRFR2 in mediating an adequate physiological, endocrine, and behavioral response to stress, followed by recent studies challenging this view. As this function is largely dependent upon structure, we will start with a short summary on the functional neuroanatomy of CRF system components followed by data on the animals’ stress (mal)adaptation response.

## Distribution of CRF System Components

### CRF

The distribution of CRF is consistent with its hypothesized functions of controlling the endocrine, physiological, and behavioral response to stress, and it is abundantly expressed in the mammalian brain, with especially high amounts of the peptide concentrated in the parvocellular division of the hypothalamic PVN, bed nucleus of the stria terminalis, central amygdala, lateral hypothalamus, and locus coeruleus (Merchenthaler et al., 1982; Morin et al., 1999). Dense CRF fibers have been located in the lateral septum, bed nucleus of the stria terminalis, central nucleus of the amygdala, median eminence, the raphe nuclei, and the spinal cord (Merchenthaler et al., 1982; Morin et al., 1999; Korosi et al., 2007).

### Urocortin 1

Urocortin 1 is related to CRF, with a sequence identify of 45% (Vaughan et al., 1995). It binds CRFR2 with 100-fold higher affinity than CRF does, indicating that this peptide might be an endogenous ligand for CRFR2 (Vaughan et al., 1995; Chalmers et al., 1996; Perrin and Vale, 1999). The most dominant sites of Ucn1 expression in the mammalian brain includes the centrally projecting Edinger–Westphal nucleus (EWcp) in the rostroventral midbrain, the supraoptic nucleus in the hypothalamus and the superior lateral olive, which have been confirmed in mammalian and non-mammalian species (Iino et al., 1997; Takahashi et al., 1998; Bittencourt et al., 1999; Kozicz et al., 2002, 2008, 2011a; Ryabinin et al., 2005). Networks of Ucn1 fibers have been identified in the lateral septum, internal layer of the median eminence, dorsal raphe nucleus, and the spinal cord, with scattered fibers located in the hypothalamus, hippocampus, cortex, and posterior pituitary (Kozicz et al., 1998; Bittencourt et al., 1999; Iino et al., 1999; Morin et al., 1999; Korosi et al., 2007).

### Urocortin 2

Urocortin 2 is a 38 amino acid peptide, which selectively binds CRFR2 (Reyes et al., 2001). Ucn2 mRNA shows a distinct subcortical distribution, including regions known to be involved in physiological and behavioral responses to stress, such as the PVN, locus coeruleus, and the arcuate nucleus (Reyes et al., 2001). Ucn2 mRNA partially overlaps with the expression of CRF in the PVN and Ucn1 in the brainstem and spinal motor nuclei (Swanson et al., 1983; Bittencourt et al., 1999; Reyes et al., 2001). To date no reliable Ucn2 immunohistochemistry has been performed (due to lack of reliable and specific antibody against Ucn2), hence the distribution of Ucn2 immunoreactive fiber terminals remains to be mapped.

### Urocortin 3

Urocortin 3 is just like Ucn2 a 38 amino acid peptide which selectively binds CRFR2 (Lewis et al., 2001). Ucn3 expressing neurons have a limited distribution compared to CRF and Ucn1, and the peptide is mostly located in forebrain regions, including the preoptic region, hypothalamus, and amygdala. Two areas of the hypothalamus express Ucn3, the first population being neurons in the preoptic nucleus and the second group of neurons are associated with the fornix (“perifornical” Ucn3 neuron population) near the PVN of the hypothalamus (Lewis et al., 2001; Li et al., 2002). More Ucn3 expressing neurons are located in the medial amygdala and paraolivary nucleus (Lewis et al., 2001; Li et al., 2002). Densest innervation of Ucn3 includes the ventromedial hypothalamus, lateral septum, posterior division of the bed nucleus of the stria terminalis and the medial amygdala, which are areas known to express CRFR2 (Lewis et al., 2001; Li et al., 2002).

### CRF binding protein

CRF binding protein is relatively widely expressed in the mammalian brain, including the cerebral cortex, subcortical limbic system, various sensory relays, raphe nuclei, hypothalamus, and pituitary (Orth and Mount, 1987; Behan et al., 1989; Potter et al., 1992). In humans, the CRF-BP has been found in the liver and in the circulation, where it inactivates CRF and/or Ucn1. The exact function of the protein is still unknown, but it has been proposed that CRF-BP prevents inappropriate pituitary-adrenal activation, e.g., during pregnancy (Potter et al., 1991). Upon binding to CRF-BP, CRF forms a dimer complex and is thought to modulate the endocrine activity of CRF (Lowry et al., 1996). In rat pituitary cells, recombinant CRF-BP blocks secretion of ACTH (Potter et al., 1991). The binding proteins have also been detected in brain regions not associated with CRF actions, suggesting it may have other functions independent from CRF (Potter et al., 1992; Cortright et al., 1995; Bale and Vale, 2004).

### CRFR1

CRFR1 is a 415 amino acid peptide in mammals and has a widespread expression in stress-related areas located in the central nervous system (CNS). CRFR1 mRNA has been located in the cortex, cerebellum, hippocampus, amygdala, olfactory bulb, lateral septum, thalamus, basal ganglia, the raphe nuclei, pituitary, brain stem, and spinal cord (Van Pett et al., 2000; Korosi et al., 2006, 2007; Justice et al., 2008; Kuhne et al., 2012). Outside the CNS, the CRFR1 is expressed in the thymus, spleen, skin, ovary, testis, gastrointestinal tissue, and adrenal gland (Dufau et al., 1993; Nappi and Rivest, 1995; Slominski et al., 1995; Baigent and Lowry, 2000; Muller et al., 2001; Chatzaki et al., 2004).

A recent study, which aimed at accurately determining the presence of CRFR1 in the brain, revealed that this receptor is present in glutamatergic neurons of the cortex and hippocampus, in gamma-aminobutyric acid containing neurons of the reticular thalamic nucleus, globus pallidus, and septum, and in dopaminergic neurons of the substantia nigra pars compacta and ventral tegmental area. It is also expressed in few serotonergic neurons of the dorsal and media raphe nuclei (Refojo et al., 2011).

### CRF receptor 2

CRF receptor 2 is a 397–437 amino acid protein in mammals and it is abundantly expressed in both the CNS and in the periphery (Kishimoto et al., 1995; Lovenberg et al., 1995b; Kostich et al., 1998; Palchaudhuri et al., 1999; Van Pett et al., 2000; Korosi et al., 2007; Justice et al., 2008; Kuhne et al., 2012). In the CNS, CRFR2 is expressed in the olfactory bulb, hippocampus, amygdala, septum, the dorsal and median raphe nuclei, cortex, pituitary, and spinal cord (Palchaudhuri et al., 1999; Bittencourt and Sawchenko, 2000; Van Pett et al., 2000; Korosi et al., 2006, 2007; Lukkes et al., 2011). In the periphery, the receptor has been identified in the retina, heart, skeletal muscle, vasculature, adrenal gland, and gastrointestinal tissue (Lovenberg et al., 1995a; Palchaudhuri et al., 1999; Muller et al., 2001; Chatzaki et al., 2004).

Despite the fact that the distribution of CRFR1 and CRFR2 overlaps in the brain, clear distinctions can be made among brain subregions. For example, the CRFR1 receptor is expressed in the basolateral nucleus of the amygdala, while both CRFR1 and CRFR2 are present in the medial nucleus of the amygdala (Bittencourt and Sawchenko, 2000; Van Pett et al., 2000). Interestingly, mRNA of CRFR2 has been discovered in non-neuronal structures as well, like the choroid plexus and cerebral arterioles (Lovenberg et al., 1995a). In the cerebral arteries and arterioles, stimulation of CRFR2 leads to an increased cerebral blood flow (De Michele et al., 2005).

## Evidence Supporting a Dualistic Action of CRFR1 and CRFR2 During Stress (Mal)Adaptation

### Stress

Stress has been defined as various physiologic alterations, including misbalance of homeostasis and activation of the hypothalamo-pituitary-adrenal (HPA)-axis (Selye, 1936, 1951; Dallman et al., 1987). Upon exposure to a stressor, the HPA-axis is activated by the release of CRF into portal vessels and subsequently acts upon the CRFR1 located in the anterior pituitary (Turnbull and Rivier, 1997; Vale et al., 1997). The initial understanding on the biological significance of CRF system components governing the endocrine stress response has come from various genetically modified animal models. As follows we will overview these animal models.

#### CRF knockout

In order to assess the central physiological role of CRF a knockout model was created (Muglia et al., 1995). Mice lacking CRF show decreased glucocorticoid levels after exposure to stress, showing the importance of CRF in regulating the HPA-axis (Muglia et al., 1995; Venihaki and Majzoub, 1999). However, interpretation of data from this animal model is significantly hampered/influenced by compensatory changes in other members of the CRF system. For example, in the absence of CRF, Ucn1 levels in the EWcp are elevated, while distribution remains unchanged (Weninger et al., 1999, 2000).

#### CRF overexpression

In order to study chronic activation of the HPA-axis, two independent CRF overexpressing mouse lines were created (Stenzel-Poore et al., 1992; Groenink et al., 2002). Both lines revealed elevated basal plasma corticosterone levels in response to stress. Delayed and attenuated HPA-axis hormone responses to stress, which might be the result of HPA-axis desensitization, were reported in the mice created by Stenzel-Poore et al. (Coste et al., 2001).

#### Ucn1 knockout

Two independent Ucn1 knockout models were generated by two groups (Vetter et al., 2002; Wang et al., 2002). Both lines have normal endocrine stress responses, supporting the notion that Ucn1 has a minor or no role in stress induced activation of the HPA-axis. It has been proposed that Ucn1 plays a role in adaptation to stress, rather than initiating the stress response. The fact that Ucn1 KO mice have impaired adaptation to repeated stress supports this notion (Zalutskaya et al., 2007).

#### Ucn2 knockout

Ucn2 knockout model showed that HPA-axis activity was normal in male and female mice deficient for Ucn2 (Breu et al., 2012). Stress induced release of corticosterone was equal between knockout and wild type animals. Interestingly, a gender specific phenotype was detected in Ucn2 deficient mice. Females, but not males, lacking Ucn2 show a significant increase in basal daily rhythms of ACTH and corticosterone (Chen et al., 2006). It is known that CRFR2 can modulate the HPA-axis, however, Ucn2 deficiency does not seem to have an impact on the stress induced activation of the axis (Breu et al., 2012).

#### Ucn3 knockout

Ucn3 knockout mouse model recently showed that absence of Ucn3 does not have an impact on basal activity of the HPA-axis, as corticosterone levels remained unchanged when compared to wild type littermates (Deussing et al., 2010). After exposure to stress, the corticosterone levels in Ucn3 knockout and wild type mice were equal, which resulted in an operational negative feedback loop of corticosterone on the HPA-axis (Deussing et al., 2010).

#### Multiple Ucn knockouts

The importance of the urocortins in the stress system was confirmed by multiple urocortin knockout studies. A double knockout of Ucn1 and 2 in mice showed equal basal corticosterone, but elevated levels after exposure to stress (Neufeld-Cohen et al., 2010a). However, the elevated corticosterone levels after exposure to stress were not observed in an urocortin 1, 2, and 3 triple knockout (Neufeld-Cohen et al., 2010b). These mice deficient for the urocortins were unable to recover properly and this was paired with dysregulated serotonergic function in stress-related neuronal circuits (Neufeld-Cohen et al., 2010b).

#### CRFR1 knockout

Two independent lines of CRFR1 knockout lines have demonstrated the importance of CRFR1 in regulation of the HPA-axis in response to stress. KO mice in both lines show an attenuated response to restraint stress by a minimal increase in ACTH and corticosterone. However, basal levels in the CRFR1 null mice are equal between KO and wild type littermates (Smith et al., 1998; Timpl et al., 1998).

#### CRFR2 knockout

CRFR2 knockout mice reveal a role for CRFR2 in regulating HPA-axis activation in response to stress, but it appears initiation of the response seems to be normal. These null mice show an early termination of ACTH release, suggesting a role for CRFR2 in maintaining activation of the HPA-axis (Coste et al., 2000). Furthermore, coping with stressors seems to be reduced in CRFR2 KO mice (Coste et al., 2000). Mice deficient for CRFR2 are also hypersensitive to stress, which leads to increased anxiety-like behaviors (Bale et al., 2000; Gammie et al., 2005). Mutant CRFR2 mice have increased CRF mRNA in the central amygdala and increased Ucn-1 and -3 in the EWcp and lateral perifornical region, respectively suggesting a compensatory activation of extrahypothalamic CRF and Ucn systems (Bale et al., 2000, 2002a; Kozicz, 2009). Chronic activation of CRFR2 also promotes an anxiety-like state, with attenuated behavioral and HPA-axis responses to stress (Neufeld-Cohen et al., 2012).

#### CRFR1 and 2 double knockout

CRFR1 and CRFR2 double knockout mice show the central role of these receptors in the stress response system. In the absence of both receptors, the double knockout mice show an impaired HPA-axis activation in response to stress (Preil et al., 2001; Bale et al., 2002b). Further, ACTH and corticosterone levels after exposure to stress are lowered in the double knockout mice compared to CRFR1 deficient mice, suggesting a role for CRFR2 in mediating HPA-axis sensitivity (Coste et al., 2000; Bale et al., 2002b).

### Anxiety

The CRF system has been proposed to be involved in the development of anxiety-related disorders. Cumulative evidence relates dysregulation of CRF systems to the etiology and pathobiology of stress-associated diseases, including anxiety. The following studies which are related to anxiety- and depressive-like behaviors are summarized in Table 1.

**Table 1:** **Summary of animal models targeting CRF system components**.

Experimental procedure	Specificity	Anxiety	Depression	Reference
**CRF KNOCKOUT**				
Targeted mutation of mice embryonic stem cells	Developmental	=	No data	Muglia et al. (1995)
**CRF OVEREXPRESSION**				
Transgenic CRF-OE mice under metallothionein promoter	Developmental	↑	No data	Stenzel-Poore et al. (1992)
Transgenic CRF-OE_2122_ mice C57BL/6J background	Developmental	No data	No data	Groenink et al. (2002)
Lentiviral based overexpression in mice	Central amygdala (1); bed nucleus of the stria terminalis (2)	(1) ↑ (2) =	(1) = (2) ↑	Regev et al. (2011)
Lentiviral based overexpression in mice	Central amygdala	↑	↑	Keen-Rhinehart et al. (2009)
**Ucn1 KNOCKOUT**				
Ucn1 gene replacement with neomycin-resistant gene cascette on C57BL/6 background (mice)	Developmental	↑	No data	Vetter et al. (2002)
Mice embryonic stem cell technology	Developmental	=	No data	Wang et al. (2002)
Mice embryonic stem cell technology	Developmental	No data	No data	Zalutskaya et al. (2007)
**Ucn2 KNOCKOUT**				
Replacement Ucn2 open reading frame by a tau-lacZ reporter gene in mice	Developmental	=	↓	Breu et al. (2012)
Ucn2 gene replacement with neomycin-resistant gene cascette on C57BL/6 background in mice	Developmental	=	↓	Chen et al. (2006)
**Ucn3 KNOCKOUT**				
Replacement Ucn3 open reading frame by a tau-lacZ reporter gene in mice	Developmental	=	=	Deussing et al. (2010)
**Ucn1 AND 2 DOUBLE KNOCKOUT**				
Crossbreeding Ucn1 and Ucn2 single knockout mice on mixed C57BL/6 × 129 background (mice)	Developmental	↓	No data	Neufeld-Cohen et al. (2010a)
**Ucn1, 2, AND 3 TRIPLE KNOCKOUT**				
Crossbreeding Ucn1, Ucn2, and Ucn3 single knockout mice on mixed C57BL/6 × 129 background (mice)	Developmental	↑	No data	Neufeld-Cohen et al. (2010b)
**CRFR1 KNOCKOUT**				
Deletion of CRFR1 locus in mice embryonic stem cells	Developmental	↓	No data	Timpl et al. (1998)
Replacement of CRFR1 gene exons by PGK-neo cascette (mice)	Developmental	↓	No data	Smith et al. (1998)
Lentiviral-based CRFR1 interference (mice)	Basolateral amygdala	↓	No data	Sztainberg et al. (2010)
Cre mediated deletion of CRFR1 (mice)	Anterior forebrain and limbic system	↓	No data	Muller et al. (2003)
Cre mediated deletion of CRFR1 in mice	Site specific knockouts in forebrain glutamatergic neurons (1); forebrain GABAergic neurons (2); midbrain dopaminergic neurons (3); brainstem serotonergic neurons (4)	(1) ↓ (2) = (3) ↑ (4) =	No data	Refojo et al. (2011)
Lentiviral based system of RNA interference in mice	Globus pallidus	↑	No data	Sztainberg et al. (2011)
**CRFR2 KNOCKOUT**				
Homologous recombination in mice embryonic stem cells	Developmental	=	No data	Coste et al. (2000)
Targeted deletion of CRFR2 locus in mice embryonic stem cells	Developmental	↑	↑	Bale et al. (2000)
CRFR2 exon replacement with neomycin-resistant gene cascette in mice	Developmental	↑	No data	Kishimoto et al. (2000)
Lentiviral based system of RNA interference in mice	Bed nucleus of the stria terminalis	↑	No data	Lebow et al. (2012)
**CRFR 1 AND 2 DOUBLE KNOCKOUT**				
CRFR2 knockout mice bred to CRFR1 knockout mice on mixed 129:C57BL/6 background	Developmental	↑	No data	Bale et al. (2002b)
CRFR2 knockout mice bred to CRFR1 knockout mice	Developmental	No data	No data	Preil et al. (2001)

*↑, indicates an increase; ↓, indicates a decrease; =, indicates no difference in anxiety- or depressive-like behavior as comparing mutant and wildtype animals*.

#### CRF knockout

Surprisingly, no behavioral abnormalities have been reported with the CRF knockouts generated by Muglia et al. (1995). Similarly, anxiety-like behavior is the same between knockout and wild type animals under normal and stressful conditions in another CRF deficient mouse created by Weninger et al. (1999). Interestingly, CRFR antagonists have an anxiolytic effect in CRF knockout animals, suggesting that CRFR1 activation is crucial to induce anxiety, while CRF itself may not. These data point toward the significance of another CRFR ligand, like Ucn1, or a yet unidentified neuropeptide, compensating for CRF deficiency in the brain (Weninger et al., 1999; Kozicz, 2007; Kozicz et al., 2011b).

#### CRF overexpression

In the model created by Stenzel-Poore et al. (1992), increased anxiety-like and decreased exploratory behavior was observed (Heinrichs et al., 1997; van Gaalen et al., 2002). This increase in anxiety-like behavior could be reversed by administration of CRF antagonist alpha-helical CRF (Stenzel-Poore et al., 1994). Removing the adrenal gland in these animals did not inhibit the anxiogenic effects of CRF overexpression, although corticosterone levels were normalized. This suggests that behavioral effects caused by CRF overexpression are mediated by CRF and CRFRs, rather than being affected by corticosterone (Heinrichs et al., 1997). Along these lines, Refojo et al. (2011) demonstrated that overexpression of limbic CRF in CRF-COE^Camk2aCre^ mice (Camk2aCre, Cre driven by the calcium/calmodulin-dependent protein kinase type II alpha chain promoter) resulted in increased anxiety-like behavior too, suggesting that limbic CRF in particular would be instrumental in mediating anxiety-like behavior. To date, no data is available on anxiety-like behavior of the CRF overexpressing mice created by Groenink et al. (2002).

#### Ucn1 knockout

The behavioral phenotype of Ucn1 KO mice is controversial. The group of Wang et al. (2002) have shown no differences in anxiety-like behavior, whereas Vetter et al. (2002) have demonstrated that Ucn1 deficient animals display increased anxiety-like behavior.

#### Ucn2 knockout

Ucn2 knockout mice have revealed no significant changes in anxiety-like behavior. However, when investigating social activities, it was seen that male Ucn2 deficient mice had more passive social interactions and reduced aggressiveness compared to wild type littermates, suggesting that Ucn2 may rather modulate aggressive behavior in male mice (Breu et al., 2012).

#### Ucn3 knockout

Ucn3 knockout mice created by Deussing et al. (2010) showed unchanged anxiety-related behaviors compared to wild type mice. However, based on the prevalent expression of Ucn3 throughout the accessory olfactory bulb and altered social discrimination abilities of male and female Ucn3 knockout mice, it has been suggested that Ucn3 plays a role in processing of social cues and establishment of social memories (Deussing et al., 2010).

#### Multiple Ucn knockout

Urocortin 1 and 2 double knockout mouse model has demonstrated an anxiolytic phenotype (Neufeld-Cohen et al., 2010a). A further reduction in anxiety-like behavior was observed after double Ucn1/Ucn2 deficient mice were exposed to acute stress, and this reduction in anxiety was correlated with levels of serotonin in anxiety-related brain regions (Neufeld-Cohen et al., 2010a). Remarkably, the triple urocortin knockout mouse showed an increase in anxiety-like behavior 24 h post-stress. This increase in anxiety in triple urocortin knockout mice was also associated with serotonergic function in stress-linked neurocircuits. It has been suggested that Ucn3 is pivotal to the observed phenotype of the triple knockout model (Neufeld-Cohen et al., 2010b).

#### CRFR1 knockout

Three studies reported that mice deficient for CRFR1 exhibited, what seemed to be, reduced anxiety-like behavior. In different tests of spontaneous anxiety, open field (a measure of exploratory behavior and general activity), light-dark box (based on the aversion of mice to well illuminated areas and on exploratory behavior in response to stressors), defensive withdrawal (a measure of conflict between exploratory behavior and retreat), and elevated plus maze (based on the aversion for open and elevated areas and on exploratory behavior in novel areas), which normally inhibit behavioral activity, CRFR1 deficient mice showed heightened levels of locomotion consistent with anxiety-like behavior (Smith et al., 1998; Timpl et al., 1998; Contarino et al., 1999). The anxiolytic effect of CRFR1 deficient mice seems to be mediated by reduced CRFR1 expression in the basolateral amygdala (Sztainberg et al., 2010). Furthermore, stress induced hormone secretion showed that CRFR1 knockout mice had reduced levels of ACTH and corticosterone, providing evidence that CRFR1 mediates anxiety and stress induced-hormone activation (Smith et al., 1998; Timpl et al., 1998). Opposite effects were found in CRFR1^Camk2aCre^ mice generated by Muller et al. (2003) in which Cre mediated deletion of CRFR1 starts in the second week of postnatal life. After exposure to restraint stress, these mice exhibited elevated levels of ACTH and corticosterone, while showing reduced anxiety-like behavior.

#### CRFR2 knockout

In contrast to CRFR1 deficient mice, CRFR2 deficient mice do not show a consistent change in anxiety-like behavior. One study has shown that CRFR2 knockouts produced no significant effects on anxiety responses in the elevated plus maze or in an open field test (Coste et al., 2000). Another study has demonstrated no behavioral changes in the light-dark test, but appealingly, the CRFR2 knockout mice have increased anxiety in the elevated plus maze and open field test (Kishimoto et al., 2000). These anxiogenic-like effects may be influenced by the reported increase in CRF mRNA in the central amygdala of the CRFR2 knockout mice (Bale et al., 2000). This brain region is associated in the activation of diverse responses induced by stress (Davis, 1992). However, some data suggests a sex effect of emotional behavior mediated by CRFR2. Female knockout mice seem to have behavior comparable to wild type littermates, while the male knockouts exhibit more anxiogenic-like behavior (Kishimoto et al., 2000).

#### CRFR 1 and 2 double knockout

CRFR 1 and 2 double knockout mice, in terms on anxiety-like behavior, displayed a sexually dichotomous phenotype (Preil et al., 2001; Bale et al., 2002b). Female double mutant mice showed reduced levels of anxiety-like behavior, while male double knockouts did not, compared to their wild type littermates (Bale et al., 2002b). The behavioral phenotype of the double knockout model generated by Preil et al. (2001) has not been researched yet.

### Depression

Strong evidence links the stress response, and the sensitivity to stressful encounters, to the development of depression (e.g., Nestler et al., 2002; de Kloet et al., 2005; McEwen et al., 2012). While the stress response is essential for successful adaptation, chronic stress can accelerate disease processes, and lead to depression or other mood disorders (Nestler et al., 2002). As follows we will highlight some of the available large body of evidence linking CRF family of neuropeptides and their receptors to the development of depression.

#### CRF

Elevated CRF levels and decreased receptor expression have been found in post-mortem examination of suicide victims. In addition, disproportionate activation of the HPA-axis has been reported in more than one-half of patients diagnosed with depression, and these symptoms can be corrected by treatment with antidepressants (Holsboer, 1999). CRF was also found to be elevated in cerebrospinal fluid of depressed patients, which was reversed in patients treated with antidepressants (De Bellis et al., 1993; Heuser et al., 1998). Reduced CRF binding sites in the frontal cortex of suicide victims have also been identified (Nemeroff et al., 1984, 1988). This was interpreted as central CRF overabundance leading to CRFR desensitization, a common phenomenon for G-protein coupled receptors (Holsboer and Ising, 2010). Furthermore, hypercortisolemia and impairment of negative feedback by cortisol on the HPA-axis have also been attributed to elevated CRF levels (Reul and Holsboer, 2002; Keck, 2006). In support for a role for CRF overexpression in the pathobiology of depression, a recent study performed by Keen-Rhinehart et al. (2009), in which CRF was chronically overexpressed in the central nucleus of the amygdala in female rats revealed an amplified CRF concentration in the PVN and a decreased glucocorticoid negative feedback, both markers which have previously been associated with the pathophysiology of depression (Nemeroff et al., 1984, 1988). Depressive-like behavior was further confirmed in the forced swim test (FST; measures escape behavior and behavioral despair), as mutant rats overexpressing CRF showed increased floating times and reduced escape behavior (Keen-Rhinehart et al., 2009).

No behavioral tests were performed on the CRF null mice created by Muglia et al. (1995) and Weninger et al. (1999) to assess depressive-like behavior.

#### Urocortins

A study where urocortin peptides have been injected i.c.v. into mice (Tanaka and Telegdy, 2008) revealed that while Ucn1 had no effect on the animal’s behavior, both Ucn2 and 3 displayed strong antidepressant-like activity by decreasing immobility time and increased climbing and swimming behavior in a FST (Tanaka and Telegdy, 2008). Moreover, single nucleotide polymorphisms (SNP) located in the gene for Ucn3 have also been associated with the antidepressant response (Wong et al., 2008). While a study assessing levels of Ucn1 and 2 has not found any significantly changes in patients suffering from major depressive disorder vs. controls (Kang et al., 2007), Ucn1 mRNA is markedly upregulated in the EWcp in depressed male suicide victims, compared to healthy males (Kozicz et al., 2008). This upregulation was not observed in female suicide victims, suggesting a gender specific neuropathology (Kozicz et al., 2008).

In animal models for depression, Ucn2 knockout mice showed a significant decrease in depressive-like behavior as assessed by the FST. These effects were only evident in the female null mice, suggesting a role for Ucn2 in mediating the sex differences observed in the stress response (Wang et al., 2002; Chen et al., 2006) albeit in an opposite direction as Ucn1 may (Kozicz et al., 2008). In contrast, mice deficient for Ucn3 have unchanged depressive-like behavior compared to wild type mice (Deussing et al., 2010).

#### CRFR1

CRFR1 activation of CRFR1 has been associated with anxiety or depressive-like behaviors, which could be treated by administrating CRFR antagonists in patients suffering from major depression (Kehne and Cain, 2010). Furthermore, quantitative PCR analyses have shown that mRNA for CRFR1, but not CRFR2, is reduced in the frontopolar cortex in suicide brains, which might be secondary to high CRF or Ucn1 levels (Merali et al., 2004; Kozicz et al., 2008). These data suggests that at least for CRFR1, dysregulation in the forebrain may contribute to the neuropathology of depression. To date, major pharmaceutical companies are focusing on CRFR1 as the primary target for antidepressant development. However, limited phase 2/3 clinical trial results with two CRFR1 antagonists, suggest a lack of efficacy in patients suffering from major depressive disorder (Kehne and Cain, 2010). This suggests that an overall inhibition of CRFR1 does not inhibit depressive-like behavior, which could mean a more complex role for CRFR1 in mediating mood disorders (see later in text).

#### CRFR2

The role of CRFR2 is more complex, as the receptor has been associated with enhancement as well as inhibition of stress responsivity (Kehne and Cain, 2010). More specifically, CRFR2 deficient mice tested in the FST displayed increased immobility which indicates depressive-like behavior (Bale and Vale, 2003; Todorovic et al., 2005) which could be effectively reversed by the CRFR1 antagonist antalarmin (Bale and Vale, 2003).

Taken together, the findings listed above have led to the notion that activation of CRFR1 is responsible for the initiation of the stress response and mediates a rather pro anxiety/depression behavior (Table 1). In contrast, CRFR2 is involved in the recovery phase of the stress response, and exhibit a more anxiolytic anti-depression function (Table 1). Although a great deal of experimental evidence supported this dualistic action of CRFR1 and CRFR2 in health and disease, not all data are in favor of this notion. Consequently, the fundamental question has recently been raised; are the endocrine, physiological and behavioral changes controlled/mediated by CRFR1 and CRFR2 the consequence of a balanced, dualistic function of these receptors or in a broader sense that of CRF system components? Or can we possibly identify specific brain regions and/or neuron populations responsible for the endocrine, physiological and behavioral stress response? Recent technical developments have allowed us to directly address this issue by creating unique animal models.

## Findings that Challenge the Dualistic Action of CRFR1 and CRFR2 in Stress (Mal)Adaptation

Conditional mutagenesis of CRFR1 using a *Cre-Lox* system has led to the development of various conditional knockouts were CRFR1 has been deleted in specific neuron populations previously implicated in stress and stress-associated anxiety and depression: (a) CRFR1^GLU-CKO^, where CRFR1 is deleted in forebrain glutamatergic neurons; (b) CRFR1^GABA-CKO^, deleting the receptor in forebrain GABAergic neurons; (c) CRFR1^DA-CKO^, carrying CRFR1 deletion in midbrain dopaminergic neurons; and (d) CRFR1^5HT-CKO^, deleting CRFR1 in brainstem serotonergic neurons (Refojo et al., 2011) (Figure 2). The roles of the different neuronal populations expressing CRFR1 on emotional behavior, these knockouts were subjected to a series of tests. CRFR1^GLU-CKO^ mice showed reduced anxiety-like behavior pointing toward a central role of glutamate neurotransmission in stress induced anxiety. These mice lack CRFR1 expression in glutamatergic neurons in the hippocampus and amygdala, two important limbic regions in the neuropathology of mood disorders (Refojo et al., 2005; Sanacora et al., 2008). In line with these data, CRFR1^Glu-CKO^ animals also show impairments in CRF-induced changes on excitatory neurotransmission in these limbic regions (Refojo et al., 2011). It was also shown that CRFR1 facilitates neuronal activity propagation from the classical hippocampal input region (dentate gyrus) to the CA1 output area. In conclusion, activation of CRFR1 in limbic glutamatergic neurons, as it would in response to a stressor, modulates glutamatergic neurotransmission, giving rise to neuronal excitation in the hippocampal network, and consequently anxiogenesis (Refojo et al., 2011).

Unexpectedly, for CRFR1^DA-CKO^, an increase in anxiety-like behavior was found, whereas for CRFR1^GABA-CKO^ and CRFR1^5HT-CKO^ no anxiety-related behavioral changes were observed (Refojo et al., 2011). As to the CRFR1^DA-CKO^, the deletion of CRFR1 in dopaminergic neurons in the midbrain ventral tegmental area and substantia nigra pars compacta (VTA/SNpc), together with the fact that CRF increases the action potential firing of dopamine neurons in the VTA (Wanat et al., 2008) are important to control the animal’s mood. It was also shown that CRFR1^DA-CKO^ displayed a decreased response to stress induced dopamine release in the prefrontal cortex (PFC), indicating that CRFR1 targets dopamine cells to control dopamine release into the PFC under stressful conditions (Refojo et al., 2011). The findings mentioned above strongly imply that stress-associated anxiety-like behavior might be a consequence of an imbalance between CRFR1 controlled glutamatergic and dopaminergic neuronal populations involved in mediating emotional behavior (Refojo et al., 2011).

In addition, the contrasting roles for CRFR1 expressed by dopaminergic vs. glutamatergic neurons suggest that under physiological conditions, CRF and CRFR1 controlled glutamatergic and dopaminergic systems might function in an antagonistic manner to maintain adaptive anxiety responses during periods of stress (Refojo et al., 2011). The fact that simultaneous deletion of CRFR1 in both glutamatergic and dopaminergic neurons do not show alterations in anxiety-like behavior, supports this notion.

A neuron population specific role for CRF system components in mediating stress-associated emotional response is further supported by a study by Sztainberg et al. (2011). These authors have demonstrated that a viral-mediated specific knock-down (KD) of CRFR1 in the globus pallidus results in increased anxiety-like behavior in mice (Sztainberg et al., 2011) (Figure 2). Earlier studies have demonstrated that this region is a key mediator of anxiety behavior, as it is associated with motor and associative functions (Baumann et al., 1999; Critchley et al., 2001; Kita, 2007; Sztainberg et al., 2011). In contrast CRFR1 expressed in the basolateral amygdala mediates anxiogenic behaviors in mice, further substantiating the view that the very same receptor can mediate opposing behavioral responses depending on the brain site of action (Sztainberg et al., 2010) (Figure 2).

To date there is only one study examining a site specific action of CRFR2. A lentiviral KD of CRFR2 specifically in the bed nucleus of the stria terminalis reduces susceptibility to stress induced anxiety in an animal model for post-traumatic stress disorder, suggesting an important role for CRFR2 in stress coping (Lebow et al., 2012) (Figure 2).

**Figure 1:**
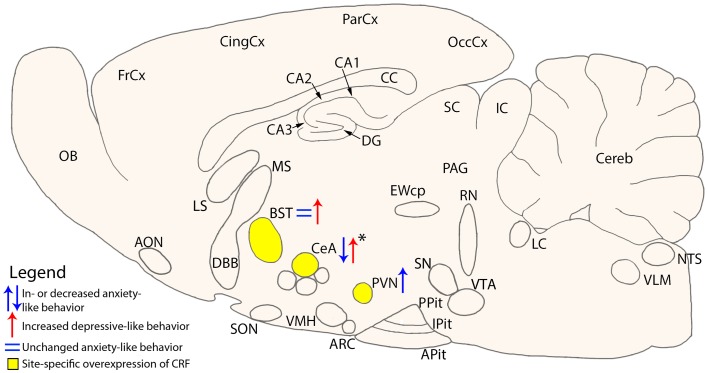
**Schematic cross-section of the mouse brain showing site specific overexpression of CRF and the resulting change on anxiety- or depressive-like behavior**. Areas of interest are the Bed nucleus of the Stria Terminalis (BST) (Regev et al., 2011), central nucleus of the Amygdala (CeA) (Keen-Rhinehart et al., 2009; Regev et al., 2011), and the paraventricular nucleus (PVN) (Elliott et al., 2010). *Change in anxiety- or depressive-like behavior is dependent on the conditions the mice were subjected to. AON, anterior olfactory nucleus; Apit, anterior pituitary; ARC, arcuate nucleus; BLA, basolateral amygdala; BST, bed nucleus of the stria terminalis; CA1-3, fields CA1-3 of Ammon’s horn; CC, corpus callosum; CeA, central nucleus of the amygdala; Cereb, cerebellum; CingCx, cingulate cortex; DBB, diagonal band of Broca; DG, dentate gyrus; EWcp, centrally projecting Edinger–Westphal nucleus; FrCx, frontal cortex; IC, inferior colliculus; IPit, intermediate pituitary; LC, locus coeruleus; LS, lateral septum; MS, medial septum; NTS, nucleus tractus solitarii; OB, olfactory bulb; OccCx, occipital cortex; PAG, periaqueductal gray; ParCx, parietal cortex; PPit, posterior pituitary; PVN, paraventricular nucleus; RN, raphe nuclei; SC, superior colliculus; SN, substantia nigra; SON, supraoptic nucleus; VLM, ventrolateral medulla; VMH, ventromedial hypothalamus; VTA, ventral tegmental area.

With regard to CRF, a site specific action can also been proposed. More specifically, prolonged and site specific overexpression of CRF in the central amygdala attenuates stress induced anxiety-like behaviors without effecting depression-like behavior, whereas CRF overexpression in the bed nucleus of the stria terminalis increased depressive-like behavior, without affecting anxiety levels in male mice (Regev et al., 2011) (Figure 1). Somewhat conflicting data were reported in a mouse model, in which unrestrained CRF synthesis in CeA produced a dysregulation of the HPA-axis, depression-like behavior, as well as physiological and reproductive consequences associated with stress-related disorders (Keen-Rhinehart et al., 2009) (Figure 1).

**Figure 2:**
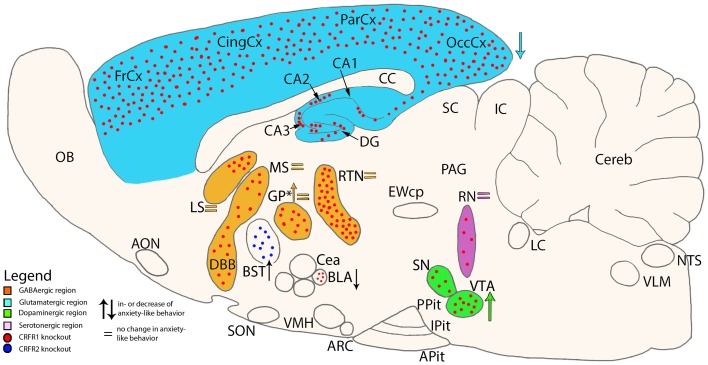
**Schematic cross-section of the mouse brain showing site specific knockouts of either CRF receptor 1 or 2 and the associated neurotransmitter specificity**. Colored arrows show the direction of change on anxiety-like behavior in mice which lack CRFR1 in specific brain areas (Refojo et al., 2011). *In one study, CRFR1 was deleted in only the GP (Sztainberg et al., 2011), while in another study CRFR1 was deleted in all areas expressing GABA (Refojo et al., 2011). Other areas of interest are the basolateral Amygdala (BLA) (Sztainberg et al., 2010) and Bed nucleus of the Stria Terminalis (BST) (Lebow et al., 2012). AON, anterior olfactory nucleus; Apit, anterior pituitary; ARC, arcuate nucleus; BLA, basolateral amygdala; BST, bed nucleus of the stria terminalis; CA1-3, fields CA1-3 of Ammon’s horn; CC, corpus callosum; CeA, central nucleus of the amygdala; Cereb, cerebellum; CingCx, cingulate cortex; DBB, diagonal band of Broca; DG, dentate gyrus; EWcp, centrally projecting Edinger–Westphal nucleus; FrCx, frontal cortex; GP, globus pallidus; IC, inferior colliculus; IPit, intermediate pituitary; LC, locus coeruleus; LS, lateral septum; MS, medial septum; NTS, nucleus tractus solitarii; OB, olfactory bulb; OccCx, occipital cortex; PAG, periaqueductal gray; ParCx, parietal cortex; PPit, posterior pituitary; RN, raphe nuclei; RTN, reticular thalamic nucleus; SC, superior colliculus; SN, substantia nigra; SON, supraoptic nucleus; VLM, ventrolateral medulla; VMH, ventromedial hypothalamus; VTA, ventral tegmental area.

Using two lentiviral-based approaches to specifically KD or conditionally overexpress (OE) CRF in the CeA of adult mice anxiety- and depression-like behaviors were evaluated under basal and stressful conditions. Intriguingly, changing CeA-CRF levels mildly affected anxiety-like behaviors under basal conditions. In contrast, following exposure to an acute stressor, CeA-CRF-KD strongly attenuated stress induced anxiety-like behaviors, whereas a short-term CeA-CRF-OE enhanced the stress induced effects on these behaviors (Regev et al., 2012). This study suggests that an adequate behavioral response to stress is not only determined by the level and site of expression of CRF system components, but depends also on the condition an animal is exposed to.

Based on the pioneering studies discussed above (for overview see Table 1 as well as Figures 1 and 2), a picture is emerging that the roles of CRF system components in the animal’s stress response and mood cannot be simplified to a dualistic model of action, but are rather linked to the recruitment of specific brain areas and neuron populations. Therefore, researches in the field must embark on a long journey to systematically and comprehensively exploring the significance of the many brain areas and neuron populations expressing one or several of the CRF system components, before we can conclude on the role(s) of CRFR1 and CRFR2 in health and disease.

## Conclusion

Taken together, recent studies using conditional mutagenesis or viral knock-down or overexpression of CRF system components strongly indicate that the involvement of CRF system components in stress-associated anxiety and depression-like behavior cannot be explained by a universal and brain wide mechanisms, and therefore the earlier proposed dualistic action of CRFR1 and CRFR2 in stress may not hold. Rather, CRF system components would be specifically recruited in particular functional neuronal circuits, which in turn would allow the expression of the necessary and sufficient endocrine, physiological, and behavioral responses to stress as conditions dictate.

## Conflict of Interest Statement

The authors declare that the research was conducted in the absence of any commercial or financial relationships that could be construed as a potential conflict of interest.
